# Quantitative trait gene *Slit2* positively regulates murine hematopoietic stem cell numbers

**DOI:** 10.1038/srep31412

**Published:** 2016-08-09

**Authors:** Amanda Waterstrat, Kyle Rector, Hartmut Geiger, Ying Liang

**Affiliations:** 1 Division of Mathematics and Natural Sciences, Somerset Community College, Laurel Campus, London, KY, USA; 2 Department of Physiology, University of Kentucky, Lexington, KY, USA; 3 Division of Experimental Hematology and Cancer Biology, Children’s Hospital Medical Center, Cincinnati, OH, USA; 4 Department of Dermatology and Allergic Disease, University of Ulm, Ulm, Germany; 5 Department of Toxicology and Cancer Biology, University of Kentucky, Lexington, KY, USA

## Abstract

Hematopoietic stem cells (HSC) demonstrate natural variation in number and function. The genetic factors responsible for the variations (or quantitative traits) are largely unknown. We previously identified a gene whose differential expression underlies the natural variation of HSC numbers in C57BL/6 (B6) and DBA/2 (D2) mice. We now report the finding of another gene, *Slit2,* on chromosome 5 that also accounts for variation in HSC number. In reciprocal chromosome 5 congenic mice, introgressed D2 alleles increased HSC numbers, whereas B6 alleles had the opposite effect. Using gene array and quantitative polymerase chain reaction, we identified *Slit2* as a quantitative trait gene whose expression was positively correlated with the number of HSCs. Ectopic expression of *Slit2* not only increased the number of the long-term colony forming HSCs, but also enhanced their repopulation capacity upon transplantation. Therefore, *Slit2* is a novel quantitative trait gene and a positive regulator of the number and function of murine HSCs. This finding suggests that *Slit2* may be a potential therapeutic target for the effective *in vitro* and *in vivo* expansion of HSCs without compromising normal hematopoiesis.

Stem cells are key to homeostatic maintenance of mature, functional cells in a variety of tissues and organs. They self-renew and produce progeny to replenish dying or damaged cells throughout an organism’s lifetime. Thus, functional failure of tissue-specific stem cells may limit tissue repair and renewal, deteriorate organismal health, and contribute to disease development^1^,^2^. The stem cells responsible for production of all blood cells are hematopoietic stem cells (HSCs), a rare cell population located in adult bone marrow. Because of the unprecedented experimental model systems that are available for exploration of HSCs, stem cell research in the field of hematology has been the subject of extensive studies^3^. It is likely that the same broad concepts defining blood-forming stem cells will apply to stem cell populations in other tissues and organs.

Stem cell regulation is a complicated and dynamic process. Identification of the collection of genes contributing to critical stem cell functions, self-renewal and multi-lineage differentiation, is far from complete. Thus, complementary strategies are needed to unravel this complex regulatory network^4^. The most widely used approach for functional analysis of specific genes is based on artificial manipulations through knockdown, overexpression or mutation in animal models. Alternatively, natural diversity and complexity of cellular traits can be linked to particular genetic variations, thus providing a powerful yet underutilized tool for the discovery of gene function^5^,^6^,^7^,^8^. This approach, proceeding from phenotype to genotype, successfully revealed genes involved in the regulation of a variety of complex traits including obesity, blood pressure, arthritis, and fatty acid metabolism^9^,^10^. Very few such genes, however, have been found in stem cells. In this study, we identified a stem cell regulatory gene accounting for the natural variation in HSC number in two mouse strains, C57/BL6 (B6) and DBA/2 (D2).

B6 and D2 mice, two commonly used inbred strains, are beneficial models for genetic mapping of phenotypic variations. We previously revealed variations in a number of HSC traits between these strains in which B6 mice have fewer HSC numbers whereas D2 mice have more. We further identified responsible quantitative trait loci (QTL) with genome-wide scans of linked genetic makers^11^,^12^,^13^,^14^,^15^,^16^,^17^,^18^,^19^. Using congenic mouse strains in which the QTL region is exchanged between two parental strains, and subsequent oligonucleotide arrays, we successfully discovered the first quantitative trait gene (QTG), *latexin (Lxn)*, and its regulatory role in HSC number and function^20^. *Lxn* expression is negatively correlated with the natural variation of HSC numbers: high Lxn level is associated with low HSC numbers in B6 mouse, whereas low Lxn expression is linked to high stem cell numbers in D2 mice. *Lxn* regulates the HSC population via a concerted mechanism of increasing stem cell self-renewal proliferation and decreasing apoptosis^20^. In an extension of this phenotypic genomic approach, several studies employed a panel of genes differentially expressed between B6 and D2 cells as a trait to map QTL that modulate gene expression (i.e., expression QTL, or eQTL)^21^. Distinct groups of eQTL, acting as either *cis*- or *trans-* controlling elements, were identified to define gene expression profiles that are specific to a single cell type and its functions, or to cellular differentiation state in a group of developmentally related cells^22^,^23^,^24^.

In this study, we employed the classic phenotypic genomic approach, and we report the finding of an additional, novel QTG, *Slit2,* which also modifies HSC number variation in B6 and D2 strains via a unique mechanism unlike that of *Lxn. Slit2* expression is positively correlated with HSC numbers and is affected by the genetic background. Increased expression of *Slit2* led to a three-fold *in vitro* and *in vivo* expansion of the HSC compartment and conferred a competitive advantage to HSCs upon transplantation. These findings may prove to be of clinical value in strategies for the effective expansion of HSCs in bone marrow transplantation for treatment of hematologic malignancies.

## Results

### Generation of chromosome 5 congenic strains

**Figure 1:**
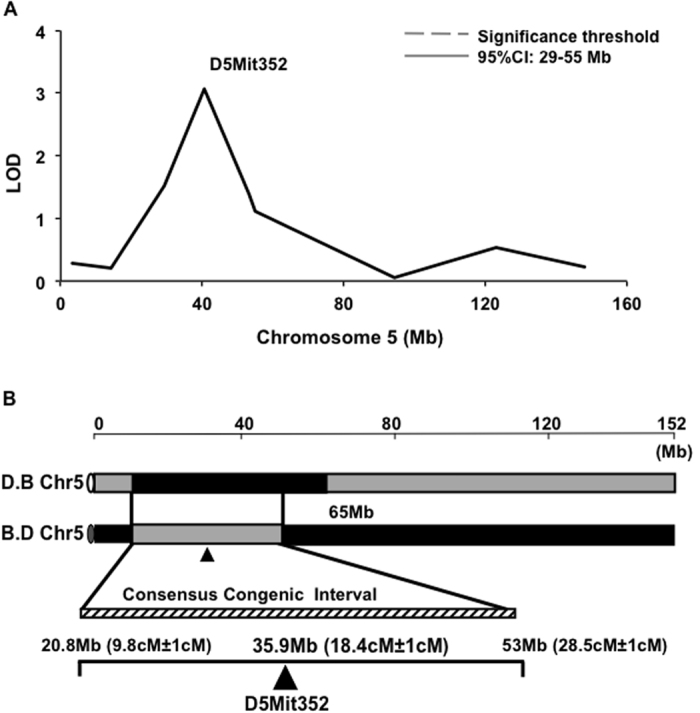
QTL mapping and genomic intervals of reciprocal Chr5 congenic mouse strains. (**A**) Linkage analysis identified Chr5 QTL as a highly associated genomic locus for HSC number variation in B6 and D2 mouse strains. Limit of detection score shows the microsatellite marker (D5Mit352) with highest association. The 95% confidence interval locates between 29–55 Mb. (**B**) Schematic illustration of genomic map of reciprocal Chr5 congenic intervals. B6-derived genomic interval harboring Chr5 QTL (black bar) was introgressed onto the D2 background (shaded bar) in congenic D.B Chr5 mice, and vice versa. The consensus congenic interval (striped bar) extends from 20.8 Mb (9.8cM ± 1cM) to 53 Mb (28.5cM ± 1cM), and includes the marker (D5Mit352) at 35.9 Mb (18.4cM ± 1cM) with the highest linkage to the trait. The non-consensus congenic interval in D.B Chr5 mice is the chromosomal region derived from B6 mice but not overlapped with B.D Chr5 line, which extends from 53 Mb to 65 Mb. The total length of Chr5 (~152 Mb) is indicated on the top.

We previously identified three QTLs that contribute to natural variation in HSC number between B6 and D2 mice. We produced congenic mouse strains for each QTL, and successfully discovered a QTG (*Lxn*) on chromosome 3 (Chr3) and confirmed its function in HSC regulation^13^,^20^. In the current study, we focus on a QTL on chromosome 5 (Chr5; Fig. 1A) and aim to unravel novel HSC regulatory mechanisms. Mouse strains congenic for the Chr5 QTL were generated by crossing the genomic interval harboring the QTL using B6 as the donor strain and D2 as the recipient strain (symbolized as D.B Chr5), and vice versa (B.D Chr5). The two reciprocal congenic strains were derived and genotyped as described previously^25^. Both congenic lines were homozygous within their respective congenic intervals. The congenic interval in Chr5 that is mutually inclusive in reciprocal strains spans from 20.8 Megabases (Mb) (9.8 centiMorgan (cM ± 1cM)) to 53 Mb (28.5cM ± 1cM), and the location of the marker (D5Mit352) most tightly linked to the trait is in 35.9 Mb (18.4cM ± 1cM) (Fig. 1B).

### Confirmation of the functional effect of Chr5 QTL in congenic strains

**Figure 2:**
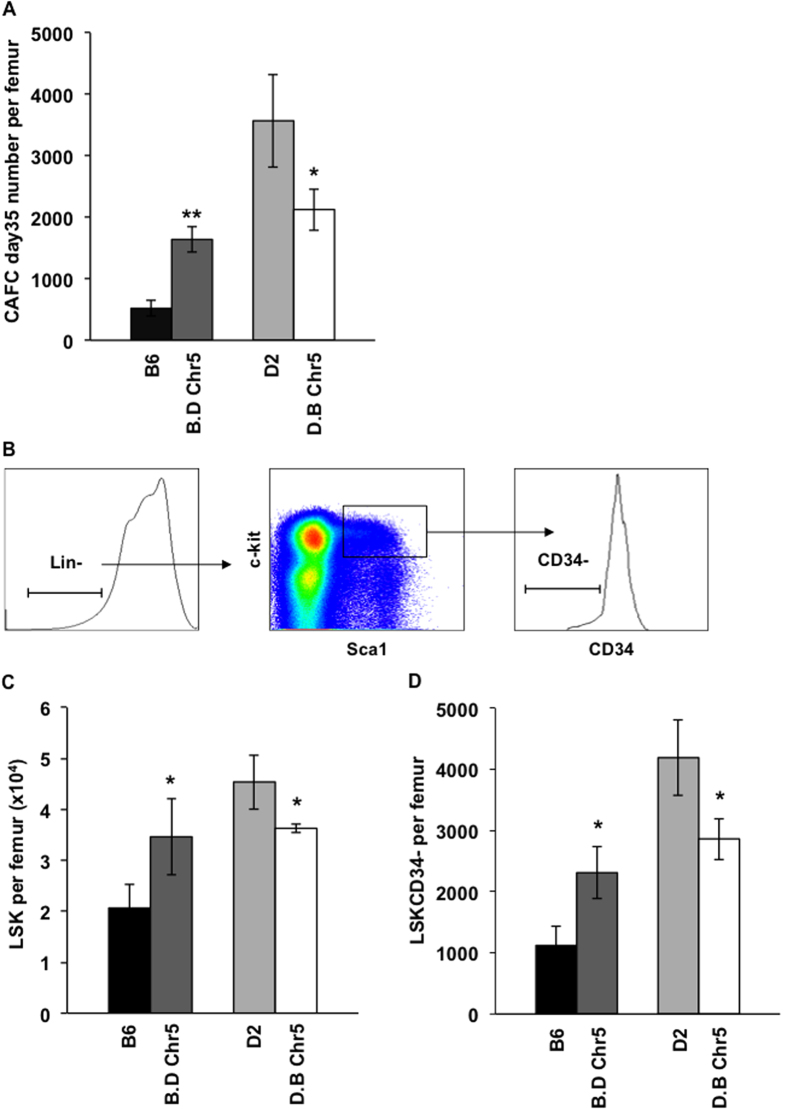
Validation of effects of Chr5 QTL on HSC number. (**A**) CAFC day 35 frequency (per femur) in reciprocal Chr5 congenic mice (B.D Chr5 and D.B Chr5) and their respective background mice (B6 and D2). The values are shown as the average of absolute number of CAFC day 35 per femur (±SEM; n > 8). (**B**) Representative flow cytometric profile for immunophenotypically-defined HSCs and HPCs. HSC/HPC are enriched in the bone marrow cells that are negative for lineage markers (Lin-) but positive for Sca-1 and c-kit markers (Sca-1+ and c-Kit+), a population referred to as LSK cells. HSCs are further enriched by negative selection of CD34 marker (LSKCD34-). (**C**) Absolute number of LSK cells per femur in reciprocal Chr5 congenic and B6, D2. (**D**) Absolute number of LSKCD34- cells per femur in reciprocal Chr5 congenic and B6, D2. Data are shown as the average (±SD) of two independent experiments (n ≥ 9). P-values were calculated using the student’s T-test with statistic significance cutoff p < 0.05. * represents p < 0.05.

To validate QTL linkage analysis, we performed a long-term *in vitro* culture assay, the cobblestone area-forming cell (CAFC) assay, on bone marrow cells derived from Chr5 congenic mice and determined cobblestone areas at day 35 of culture, which is a proxy for the number of long-term HSCs that were present in the seeded bone marrow cells. Introgression of D2 alleles onto a B6 genetic background in the B.D Chr5 congenic line led to a three-fold increase in HSC number as compared to B6 background (Fig. 2A), whereas transfer of B6 alleles onto the D2 genetic background decreased HSC number by 50% in the D.B Chr5 strain. Moreover, cell counts for peripheral blood leukocytes, erythrocytes and platelets, and numbers for CAFC days 7 and 21, which represent hematopoietic progenitor cells at different stages, showed no difference between Chr5 congenic and their respective parental strains, except for CAFC day 21 between D2 and D.B Chr5 mice (Suppl. Table 1), suggesting that the non-consensus congenic region in D.B Chr5 (53 –65 Mb, see Fig. 1B) mouse may affect hematopoietic progenitor cell (HPC) number. To further confirm the CAFC result, we quantified the immunophenotypic HSCs, which are defined as cells negative to lineage markers (Lin-), positive for Sca-1 and c-kit (Sca-1+ and c-Kit+), and negative for CD34 (CD34-) (Fig. 2B). Consistently, the result showed that B.D Chr5 mice had more HSC/HPC-enriched Lin-Sca-1 + c-Kit+ (LSK) cells (Fig. 2C) and HSC-enriched LSKCD34- cells (Fig. 2D) than B6 mice, whereas reciprocal D.B Chr5 mice had fewer cells in these two populations compared to D2 mice. Altogether, these results suggest that Chr5 QTL specifically regulates the size of the HSC population, and that the D2 allele is associated with an increased stem cell number whereas the B6 allele is associated with decreased stem cell number.

### Chr5 QTL enhances HSC repopulation capacity in a cell-intrinsic manner

**Figure 3:**
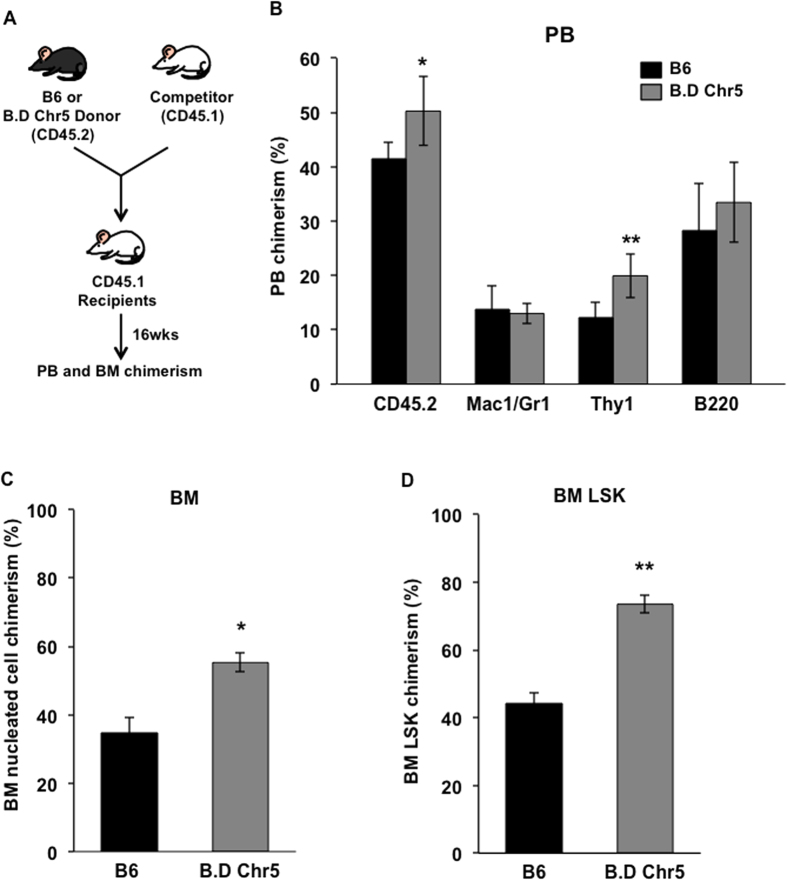
Increased repopulation capacity of B.D Chr5 congenic bone marrow cells. (**A**) Competitive repopulation assay. Donor bone marrow cells (B6 or B.D Chr5 mice) were transplanted into the lethally irradiated recipient mice along with an identical number of competitor bone marrow cells. The CD45 marker is used to distinguish donor cells from competitor and recipient mice-derived cells, which are CD45.2 and CD45.1, respectively. The competitive repopulation capacity of donor cells is determined by CD45.2/CD45.1 chimerism in peripheral blood (PB) and bone marrow (BM) of recipients at 16 weeks post-transplant. (**B**) Donor contribution to leukocytes (CD45.2) and all blood lineages in PB of recipients. Macrophages and granulocytes are labeled by Mac1/Gr1 marker. T and B lymphocytes are labeled by Thy1 and B220, respectively. (**C**) Donor contribution to bone marrow nucleated cells of recipient mice. (**D**) Donor contribution to bone marrow LSK cells of recipient mice. Data are shown as the average (±SD) of two independent experiments (n = 10). P-values were calculated using the student’s T-test with statistic significance cutoff p < 0.05. * represents p < 0.05. ** represents p < 0.01.

To determine whether Chr5 QTL can intrinsically regulate HSC number and function, we performed the competitive repopulation assay, in which B6 or B.D Chr5 bone marrow cells were transplanted into the same recipients (or environment) and repopulated their blood system (Fig. 3A). B.D Chr5-derived cells made a greater contribution to reconstitution of peripheral blood leukocytes, especially T lymphocytes, total bone marrow cells, and HSC/HPC-enriched LSK cells (Fig. 3B–D), suggesting that D2 QTL enhances HSC repopulation capacity. Moreover, The increased level of LSK cells in B.D Chr5 cells reconstituted recipients was similar to that in B.D Chr5 congenic mice (Fig. 2C), indicating the cell-intrinsic role of this congenic interval. It should be noted that the recipient mice carrying CD45.1 markers are not commercially available on D2 genetic background, which precluded us from performing competitive repopulation assay on D2 and D.B Chr5 bone marrow cells. We further determined the cell cycle and apoptotic status of congenic and background HSCs because both cellular mechanisms contribute to maintenance of the HSC pool. No differences in percentages of cycling and apoptotic cells were detected in congenic strains compared to their respective background strains (B.DChr5 vs B6, and D.BChr5 vs D2) (Supp. Fig. 1A,B). However, a significantly higher percentage of LSKCD34- HSCs underwent apoptosis in mice with B6 background than those with D2 background (Supp. Fig. 1B), suggesting the effect of genetic background on the function of HSCs. Altogether, these results imply that Chr5 QTL and underlying QTGs are involved in the regulation of repopulation and multi-lineage differentiation of HSCs in a cell-intrinsic manner.

### Identification of candidate QTGs by microarray and quantitative polymerase chain reaction (PCR)

**Figure 4:**
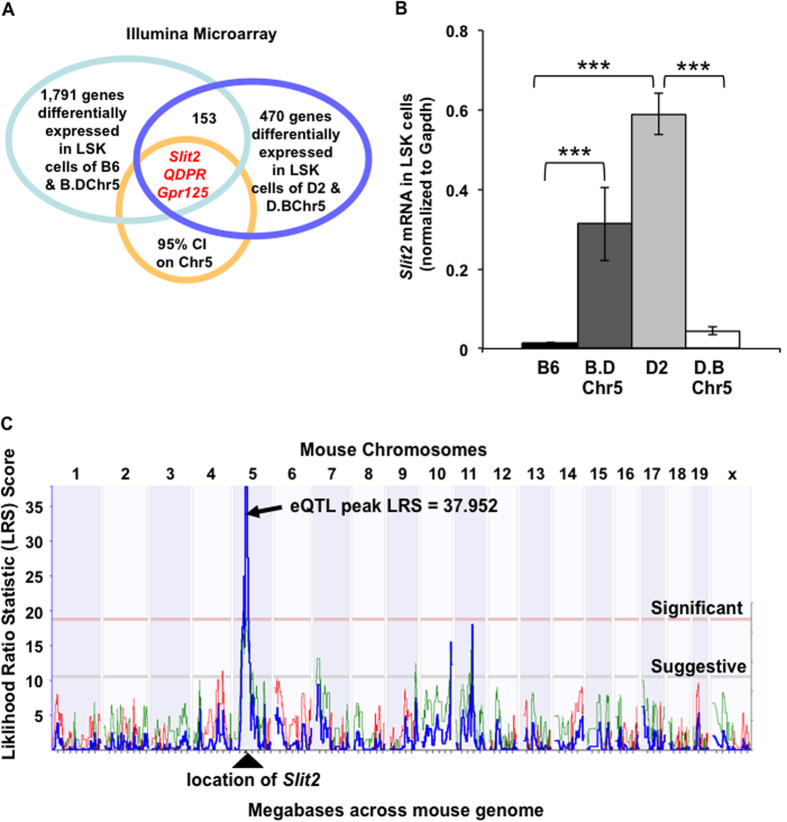
Differential *Slit2* expression in HSC/HPCs of Chr5 congenic and background mice. (**A**) Microarray analysis of gene expression in LSK cells revealed 1,791 differentially expressed genes between B6 vs B.DChr5 mice, and 470 differentially expressed genes between D2 and D.BChr5 (GEO#:GSE21896). In the two Illumina microarray data sets, 153 differentially expressed transcripts were common between the two comparisons. Three transcripts were located within the 95% confidence interval for the Chr5 QTL. (**B**) *Slit2* mRNA expression in HSCs. Identical number of LSK cells were sorted from each strain and *Slit2* mRNA level in these cells was quantified by real-time PCR (normalized to endogenous control GAPDH expression). *Slit2* is expressed at significantly higher levels in the LSK population of mice bearing D2 alleles at the Chr5 QTL position (***p < 0.001). Results are the average (±1 SD) of 8–12 measurements derived from two to three independent biological samples. Statistical significance of strain-specific expression differences was determined using the 2-tailed T-test. (**C**) *Slit2* expression is cis-regulated. Linkage analysis was performed on GeneNetwork to search for the eQTL that influences *Slit2* expression. It revealed a highly significant eQTL (LRS = 38 on Chr5; blue line). The entire mouse genome is labeled across the x-axis (Mb). The LRS score is indicated on the y-axis. The positive additive coefficient (solid green line) indicates that D2 alleles increase trait values.

In order to investigate QTG underlying Chr5 QTL, we obtained gene expression profiles on HPC/HSC-enriched LSK cells of B6, B.DChr5, D2, and D.BChr5 strains using the Illumina microarray platform. We revealed 1,791 differentially expressed genes between B6 and B.DChr5 HSCs and 470 differentially expressed genes between D2 and D.BChr5 cells (GEO Accession # GSE21896), in which 153 genes overlapped between the two comparison groups (Fig. 4A). We screened these genes on the basis of genomic location within the 95% confidence interval (20.8–53 Mb) surrounding the Chr5 QTL and identified three candidate genes: *Slit2, QDPR* and *Gpr125*. Quantitative real-time PCR confirmed that only *Slit2* showed reproducible patterns of differential expression in LSK cells in both congenic-parental strain comparisons (Fig. 4B). The presence of D2 alleles in the congenic interval resulted in a high level of *Slit2* expression relative to almost undetectable levels in mice bearing the B6 genotype. A roughly 30-fold increase in *Slit2* expression was observed in B.D Chr5 LSK cells relative to B6 and a similar fold decrease in D.BChr5 LSK cells relative to D2. These data suggest that *Slit2* expression is positively correlated with HSC numbers, and its expression pattern in HSCs is dramatically affected by genetic background.

### eQTL is associated with the differential expression of Slit2 between B6 and D2 mice

Differential expression of *Slit2* between both congenic and parental strains suggests that *Slit2* expression is governed by a polymorphic regulatory region within the congenic interval. Consistent with this observation, 3 consecutive, highly significant eQTL were identified for the *Slit2* transcript in HSCs using the “genetical genomics” approach (http://genenetwork.org/webqtl/main.py). The peak eQTL (Likelihood Ratio Statistic = 37.952) corresponds to 13 consecutive markers spanning 45.6–53.1 Mb on Chr5 (Fig. 4C), and encompasses the entire *Slit2* transcript (48.376032–48.696750 Mb). In addition, the overlapping green line in the eQTL peak suggests that the D2 allele is associated with high *Slit2* expression, which corroborates our microarray and real-time PCR results. Our discovery of *Slit2* differential expression in B6 and D2 LSK cells and the search for responsible eQTL are consistent with results by Gerrits *et al*., which also identified distinct specificity of *Slit2* eQTL in a stem cell population^23^. These results suggest that the *Slit2* gene is *cis*-regulated and the single nucleotide polymorphisms flanking the *Slit2* gene most likely contribute to its differential expression in B6 and D2 HSCs.

### Ectopic Slit2 expression results in expansion of functional HSCs

**Figure 5:**
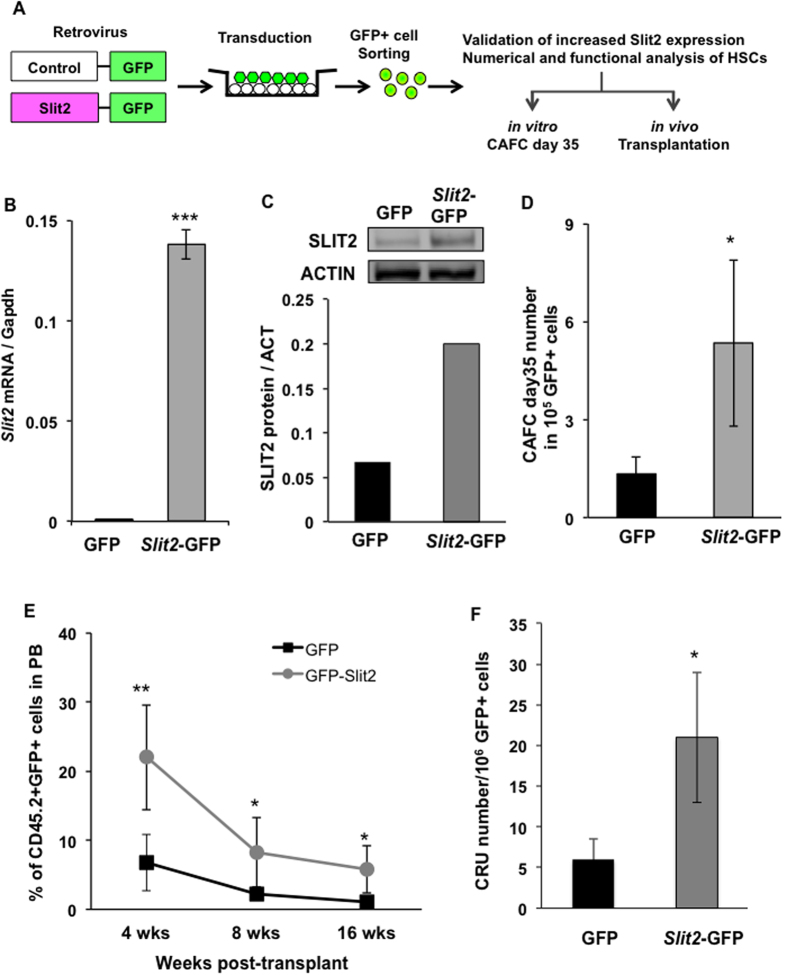
Ectopic expression of *Slit2* increases HSC number and repopulation function. (**A**) Retrovirus-mediated overexpression of *Slit2* in B6 HSC/HPCs and its effect on HSC number and function. (**B**) Enhanced *Slit2* mRNA expression in transduced bone marrow cells. B6-derived bone marrow cells were transduced with GFP-bearing retroviral vectors with or without *Slit2 (Slit2*-GFP or GFP control). GFP+ marrow cells were flow cytometrically isolated and their *Slit2* expression was quantified by real-time PCR. ***p < 0.001. (**C**) Enhanced *Slit2* protein expression in transduced bone marrow cells. (**D**) *Slit2* overexpression increases CAFC day 35 number. GFP+ cells were isolated by flow cytometry and were evaluated by CAFC assay. Cells that can form colonies at day 35 of cell culture were quantified and compared between *Slit2*-GFP and GFP control. Data shown are the average of two independent experiments in two separate biological samples (n = 10). *p < 0.05. (**E**) *Slit2* overexpression increases the competitive repopulation capacity of HSCs. *Slit2*-overexpressing HSC/HPCs were transplanted into lethally irradiated recipient mice. GFP+ donor-derived blood leukocytes (CD45.2+) were monitored for 16 weeks. Data shown are the average of two independent experiments in two separate biological samples (n = 10). (**F**) *Slit2* overexpression increases the number of long-term repopulating cells. Graded numbers of *Slit2*-overexpressing GFP+ cells were transplanted into lethally irradiated recipient mice. GFP+ donor-derived blood leukocytes in each blood lineage was examined at 12 weeks post-transplant. *p < 0.05. **p < 0.01.

Having established a positive correlation between *Slit2* expression and HSC numbers under physiologic conditions, we enforced *Slit2* expression in HSC/HPCs and determined its direct regulatory role in HSC number and function. Using the same overexpression strategy as previously published^20^, we cloned the *Slit2* cDNA into a retroviral vector containing the green fluorescent protein (GFP) reporter, and overexpressed *Slit2* in HSC/HPCs from B6 mice, which normally express little or no *Slit2*. HSC number was measured by CAFC assay on GFP positive bone marrow cells (GFP control or *Slit2*-GFP) (Fig. 5A). The increased expression of *Slit2* at mRNA and protein levels was confirmed by real-time PCR and Western blot, respectively, in *Slit2-*GFP cells compared to control GFP cells (Fig. 5B,C). Overexpression of *Slit2* resulted in a 3-fold increase in the number of HSCs (CAFC day 35) (Fig. 5D), strongly suggesting *Slit2* as a positive regulator of HSC number. In order to determine *in vivo* function of *Slit2* in hematopoiesis, we transplanted 2 × 10^5^ *Slit2*-GFP or control GFP cells along with the same number of competitor cells (a standard approach in murine bone marrow transplantation studies to ensure recipient survival) into lethally irradiated recipient mice and measured reconstitution of GFP+ cell in peripheral blood at different time-points post-transplant. *Slit2*-overexpressing cells (*Slit2*-GFP) made a greater contribution to blood reconstitution at both short-term (4 weeks and 8 weeks) and long-term (16 weeks) post-transplant (Fig. 5E), demonstrating that *Slit2* can increase the repopulating capacity of HSCs. This result is consistent with that seen in B.D Chr5 congenic HSCs, which have naturally high levels of *Slit2* expression (Fig. 3B). To further confirm the positive regulatory role of *Slit2* in HSC numbers, we performed a more stringent assay (i.e., long-term limiting-dilution analysis in competitively repopulated hosts) to functionally identify and quantify HSCs *in vivo*. We found that overexpression of *Slit2* caused a 3-fold increase in the number of long-term repopulating HSCs (Fig. 5F). Thus these results robustly suggest *Slit2* as a positive regulator of HSC number and function.

## Discussion

**Figure 6:**
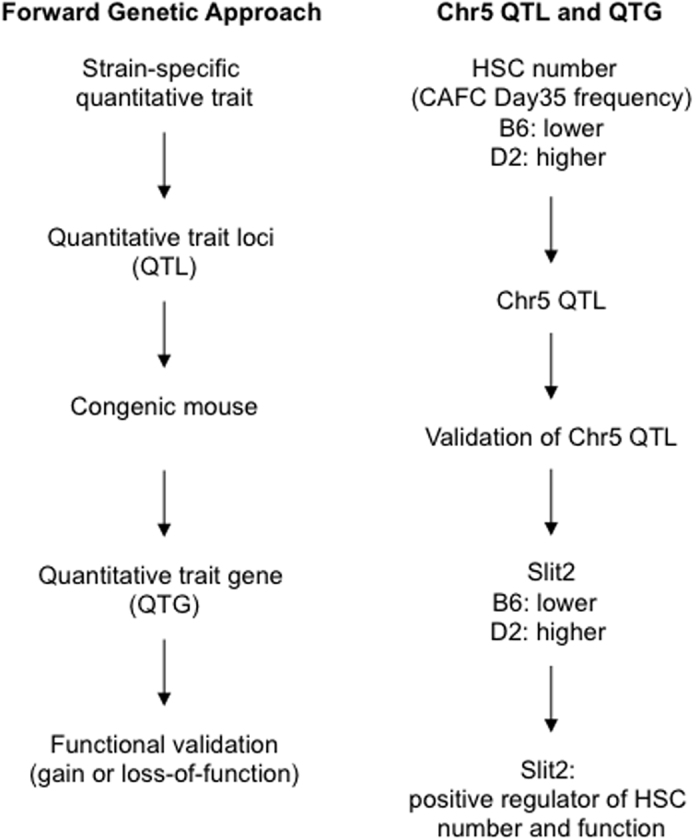
Forward genetic approach leads to the discovery of *Slit2* as a quantitative trait gene. Forward genetic approach begins with strain-specific quantitative trait. Linkage analysis identifies quantitative trait loci (QTL) that are responsible for the trait. Generation of congenic mouse strains in which the QTL are exchanged between parental strain will facilitate the confirmation of QTL and their functional effect. Gene expression profiles will identify the underlying quantitative train genes (QTGs). The regulatory role of QTGs in quantitative trait needs to be further confirmed by gain or loss of gene function. By following this pathway, our study identified Chr5 and underlying *Slit2* as QTG that accounts for the natural variation in HSC number between C57BL/6 (B6) and DBA2 (D2) mouse strains. *Slit2* expression level positively correlates with HSC number. *Slit2* level is lower in B6 mice that have less HSC number whereas its level is higher in D2 mice that have more HSCs. Ectopic expression of *Slit2* leads to the increased HSC number and function in B6 mice, further confirming *Slit2* as a positive regulator of HSCs.

In this study, we identified a new quantitative trait gene, *Slit2*, as the positive regulator of HSC number and function. We used the QTL mapping method, which is a powerful approach for discovering genetic determinants of complex traits^8^. We began with the observation of natural HSC number variation between two genetically distinct mouse strains (Fig. 6): the B6 strain has lower stem cell numbers and the D2 strain has higher numbers. Genetic mapping analysis identified a locus on chromosome 5 that is associated with this natural variation. We then generated reciprocal congenic mouse strains in which the genomic locus harboring the QTL was exchanged between the B6 and D2 strains. Quantification of HSC number in these congenic strains confirms that the chromosome 5 QTL and the candidate gene(s) must not only be located within the congenic interval but, if functioning at the level of the transcript, the regulatory element governing expression must also be present in the congenic interval. The congenic mice were also particularly valuable in microarray analyses to identify candidate genes. Among the 2,261 unique differentially expressed transcripts identified in the Illumina microarray experiment, only 153 were common between the two congenic/background strain comparisons, representing a 94% reduction in the number of genes differentially expressed between the B6 and D2 parental strains alone. This reduction greatly facilitated our selection of *Slit2* as a candidate gene because it is the only differentially expressed gene that is present in the consensus congenic interval. Therefore, given the novel role for *Slit2* in the hematopoietic system, we benefited greatly from the ability of the QTL mapping approach to eliminate bias introduced by selecting genes on the basis of *a priori* knowledge about function. Elimination of *Slit2* in knock-out mice results in embryonic lethality^26^. Unlike mutagenesis techniques, lethality or nonspecific effects are not hampered by QTL-based gene discovery, thus preventing detection of a phenotypic effect on adult tissue.

The mechanisms by which *Slit2* regulate hematopoiesis have not been identified. However, several recent publications have reported the function of its binding partner, *Robo4,* in HSC regulation^27^,^28^. Smith-Berdan and colleagues unraveled the important role of *Robo4* in anchoring HSCs to bone marrow niche. Loss of *Robo4* specifically decreased the number of HSC in bone marrow and impaired bone marrow engraftment capability. Interestingly, absence of *Robo4* was compensated with up-regulation of *CXCR4*, which led to less efficient mobilization of HSCs from bone marrow to the periphery. The authors also investigated the role of *Slit2* in *Robo4* function and found that *Slit2* didn’t affect HSC proliferation and migration *in vitro*. The study by Shibata *et al*. showed that *Slit2* was specifically expressed in bone marrow stromal cells of the B6 strain, but not HSCs, and overexpression of *Slit2* led to the differentiation of primitive HSCs to less-primitive marrow cells. In addition to HSCs, a recent study has shown that *Slit2* transgenic mice have increased intestinal stem cell numbers^29^. Our findings are in agreement, at least partly, with these literature reports. We found that *Slit2* and the *Slit2*-containing Chr5 QTL do not influence HSC proliferation and apoptosis (Supp. Fig. 1). Like intestinal stem cells, overexpression of *Slit2* led to the increased number of functional clonogenic and repopulating HSCs (Fig. 5). More importantly, our study demonstrates for the first time the strain-specific difference in *Slit2* expression in physiological conditions. Indeed, the function of *Slit2* in HSCs seems to be overlooked due to the fact that very little or no detectable *Slit2* transcript was present in HSCs of the B6 mouse strain compared to the D2 strain (Fig. 4B). This observation not only explains the reason that *Slit2* was not detected in HSCs in Shibata’s study, but also reveals the importance of genetic background in modification of critical stem cell regulatory genes. It would be intriguing to employ the D2 mouse strain as a model to investigate *Slit2* function in the regulation of HSCs and stromal cells in future studies.

The role of *Slit2* as a secreted protein in governing cellular migration, spatial orientation, organogenesis and development in other tissues is well known^30^,^31^,^32^,^33^,^34^. Here, we provide strong evidence to support the cell-intrinsic role of *Slit2* in increasing HSC numbers. In competitive repopulation assays in which B6 or B.D Chr5 cells were injected into the same environment (recipients), B.D Chr5 showed an advantage in repopulating blood and bone marrow stem/progenitor cells (Fig. 3). Moreover, *Slit2* overexpressing cells showed a similar increase in their repopulating capacity and in the absolute number of repopulating cells upon transplantation (Fig. 5E,F). Such changes are similar to what we observed in the B.D Chr5 congenic mice *in situ*, strongly supporting that *Slit2* plays an intrinsic role in regulating HSC function. However, because *Slit2* is also highly expressed in the bone marrow niche cells, such as osteoblast and mesenchymal stem cells^35^, we cannot exclude the possibility that *Slit2* regulates HSC numbers by influencing stem cell-niche interaction. It is possible that high *Slit2* expression may result in preferential localization in niches that favor stem cell self-renewal, and low *Slit2* expression results in occupation of niches that favor differentiation^36^,^37^,^38^. In addition, compensatory alterations in other signaling pathways, such as *CXCR4,* may account for important differences in HSC number and functions between B6 and D2 mice. Further experiments, such as reciprocal transplantation with B6 or B.D Chr5 mice as recipients, will be interesting to determine the cell-intrinsic and/or cell-extrinsic role of *Slit2* in regulating HSC function.

Our previous studies identified *Lxn* on the Chr3 QTL as a negative regulator of HSC number, and Chr3 and Chr5 QTLs as contributing loci for the natural variation of HSC number in B6 and D2 mouse strains^20^. In combination with discovery of *Slit2* as a positive regulator herein, it will be of great interest to investigate synergetic and/or additive roles of these genes in homeostatic maintenance of the HSC population size. It may unravel a novel signaling pathway for HSC regulation. Although no evidence functionally links *Lxn* and *Slit2*, a recent study showed that *Lxn* is co-localized with *Robo4* in hematopoietic stem/progenitor cells, and its ablation reduces the abundance of *Robo4* protein^39^. We speculate that loss of *Lxn* expands the HSC population and HSCs reflexively down-regulate *Robo4* expression and decease HSC numbers. Thus, *Lxn* and *Slit2/Robo4* signaling pathways may counteract each other and cooperatively control HSC population size in normal physiological conditions. We are currently investigating this hypothesis. If it can be experimentally supported, it highly underscores the importance and power of the QTL approach in uncovering a genetic regulatory network in stem cell function. Uncovering the genetic networks that govern natural variation in complex traits such as stem cell numbers make it possible to identify a specific group of regulatory genes as well as their cooperative roles in determining stem cell functions. It would be difficult to appreciate the complexity of such networks, governing a homeostatic mechanism like the maintenance of hematopoiesis, without the QTL to QTG strategy.

In summary, we report compelling evidence for the identification of *Slit2* as a QTG on mouse Chr5 that influences HSC numbers in mice. Gene expression analysis of primary hematopoietic cells, overexpression, and functional studies reveal a novel role for *Slit2* as a positive regulator of HSC numbers. It is likely that polymorphisms between B6 and D2 in the *Slit2* or a nearby *cis* regulatory element are responsible for strain-dependent differential expression. These findings may lead to effective strategies for *in vitro* and *in vivo* expansion of the HSC population, enhancement of HSC mobilization and engraftment, and improved strategies for HSC transplantation therapy.

## Methods

### Animals

Six to 16 week old female C57BL/6J(CD45.2), B6.SJL/J(CD45.1) and DBA/2J mice were purchased from The Jackson Laboratory (Bar Harbor, ME) and congenic mice generated by our laboratory were used in all experiments. Mice were given food and acidified water *ad libitum* and housed in pathogen free conditions in the animal facility of the University of Kentucky and were maintained according to NIH guidelines for animal welfare. All animal and experimental procedures were approved by IACUC and IBC offices of the University of Kentucky with protocol numbers 2012-0999 (IACUC) and B13-2133 (IBC).

### Generation of Chr5 congenic strains

Congenic strains were generated by selective, marker-assisted breeding as previously described^25^. The genomic interval carrying the QTL from B6 was transferred onto a D2 genetic background to generate the D.B Chr5 congenic strain. The reciprocal congenic, designated B.DChr5 was generated by transfer of the D2 QTL region onto the B6 background. Both strains were bred to homozygosity at all loci, as verified by genotype analysis with 100 microsatellite markers distributed across the mouse genome. The consensus congenic interval ranges from 20.8–53 mega bases on Chr5, encompassing the entire 95% confidence interval identified by linkage analysis.

### Peripheral blood cell counts

Anesthetized mice were bled from the retro-orbital venous plexus. Circulating leukocyte, erythrocyte and platelet counts were measured by analysis of 40 ul blood using a System 9118^+^ Hematology Series Cell Counter (Biochem Immunosystems, Allentown, PA).

### Cobblestone area-forming cell assay

CAFC assay was performed as previously described to quantify hematopoietic stem and progenitor cells^20^. We performed simultaneous analysis of CAFCs in B6, D2, and congenic strains as well as in *Slit2*-GFP vs GFP control cells. In brief, a confluent monolayer of FBMD-1 stromal cells was established in 96-well tissue culture-treated plates (Costar, Cambridge, MA). After 7 to 10 days, wells were seeded either with unfractionated marrow or GFP+ cells at a dose of 81,000, 27,000, 9,000, 3,000, 1,000, or 333 cells per well. Twenty replicate wells per cell number were evaluated. The cells were cultured in Iscove’s Modified Dulbecco Medium, containing 20% horse serum, 80 U/mL penicillin, 80 mg/mL streptomycin (all from Life Technologies), 10^−4^ ß-mecaptoethanol, and 10^−5^ M hydrocortisone (both from Sigma, St. Louis, MO). Individual wells were screened at days 7, 14, 21, 28 and 35 for the presence of a cobblestone area, defined as a colony of at least 5 small, non-refractile cells growing underneath the stroma. The longer the latency before cobblestones appear, the more primitive the cells forming that cobblestone. Thus, the most primitive HSCs show cobblestones at day 35, whereas colonies that appear earlier are derived from more committed progenitor cells. Frequencies of CAFCs were calculated by using maximum likelihood analysis and equals 1 divided by the number of cells yielding 37% negative wells. Statistical comparisons were performed using the L-Calc software package from Stem Cell Technologies (Vancouver, Canada). Statistically significant difference between populations being compared was determined using a p-value threshold of 0.05.

### Immunofluorescent staining and sorting of hematopoietic stem cells

Bone marrow cells suspended in Hank’s balanced salt solution (HBSS, Gibco, Grand Island, NY) containing 2% fetal calf serum (FCS) (Life Technologies) were blocked with anti-CD16/32 (clone 2.4G2, Fc Block) to prevent non-specific binding. Cells were stained with the lineage markers, including CD5 (clone 53-7.3), CD8a (clone 53-6.7), CD45R/B220 (clone RA3-6B2), CD11b/Mac-1 (clone M1/70), Ly-6G/Gr-1 (clone RB6-8C5), TER119/Ly-76 (clone TER-119), and stem cell-specific markers, Ly-6A/E (Sca-1; clone E13-161.7), CD117 (c-kit; clone2B8) and CD34 (clone RAM34). The viable cells were distinguished by their ability to exclude propidium iodide (5 ug/mL). All monoclonal antibodies were purchased from Pharmingen, San Diego, CA. Flow cytometric analysis and sorting was performed on a FACSAriaII (Becton Dickinson Immunocytometry Systems, San Jose, CA) to select propidium iodide negative, lineage negative, Sca-1 and c-kit positive, and CD34 negative cells that are highly enriched in viable HSCs.

### Cell cycling and apoptosis

Bone marrow cells suspended in HBSS (Gibco, Grand Island, NY) containing 2% FCS (Life Technologies) were blocked with anti-CD16/32 (clone 2.4G2, Fc Block) to prevent non-specific binding. Cells were labeled as described in the immunofluorescence staining section. Cells were fixed and permeabilized in BD Cytoperm/CytoFix buffer (BD Bioscience). Subsequent co-labeling with Ki-67 and DAPI enabled the determination of cell cycle stage and DNA content. Cells negative for Ki-67 and DAPI are in G0 phase, cells positive for Ki-67 but negative to DAPI are in G1 phase, and cells positive for both markers are in S/G2/M phases of cell cycle. Apoptotic cells were stained with Annexin V and 7-AAD as previously described^20^. Briefly, bone marrow cells were prepared and immunofluorescently stained as described above. Fluorescein isothiocyanate-conjugated Annexin V and 7-AAD were used to identify apoptotic cells, which are Annexin V positive and 7-AAD negative. Thymocytes were used as the control to distinguish Annexin V-positive and -negative cells (Pharmingen, San Diego, CA). Three independent experiments were performed in each strain, and at least 3 mice were analyzed in each experiment. All experiments were carried out on ice and completed within 1.5 hours. Flow cytometric analysis was performed on a triple-laser FACSAriaII (Becton Dickinson Immunocytometry Systems, San Jose, CA). All antibodies used in this assay were purchased from BD Pharmingen (NJ).

### Competitive repopulation assay

1 × 10^6^ donor cells were mixed with an equal number of competitor bone marrow cells (B6.SJL/BoyJ) and retro-orbitally injected into lethally irradiated (9 Gy) recipient mice. Donor cells were either derived from B.D Chr5 congenic or B6 mice (CD45.2), or derived from B6 bone marrow cells with or without *Slit2* overexpression (GFP-*Slit2* or GFP+) cells. Recipients were bled from the retro-orbital sinus at 4, 8 and 16 weeks post-transplantation. Erythrocytes were depleted by hypotonic lysis using NH4Cl. The leukocytes were stained in triplicate with fluorescein isothiocyanate-conjugated anti-CD45.2 monoclonal antibody (clone ALI4A2) and phycoerythrin-conjugated monoclonal antibody (Becton-Dickinson-PharMingen, San Diego, CA) specific for either B (anti-CD45R/B220; clone RA3-6B2) or T lymphocytes (anti-Thy-1.2; clone 30H12), or granulocytes (anti-Ly6G/Gr-1; clone RB6-8C5) and macrophages (anti-CD11b/Mac-1; clone M1/70). Percentages of donor (CD45.2, or GFP+)-derived PB were analyzed using a FACScan instrument (Becton-Dickinson Immunocytometry Systems, San Jose, CA). At 16 weeks post-transplant, bone marrow cells were harvested, stained for LSK cells as described above, and then analyzed for donor (CD45.2 or GFP+)-derived cells in FACSAriaII.

### RNA isolation

Total RNA was extracted from hematopoietic cells using the Quiagen RNeasy Mini Kit (Qiagen, Valencia, CA) according to manufacturer’s instructions. This isolation method selects total RNA using silica-membrane RNeasy spin columns. Isolated RNA was eluted in water and either used directly for microarray analysis or reversed transcribed to cDNA for quantitative real-time PCR.

### Microarray analysis

Triplicate RNA samples were isolated from 80,000–137,000 LSK cells of B6, D2, B.DChr5, and D.BChr5 mice. Each of the 12 samples was reverse transcribed, purified, labeled, and hybridized to 12 individual arrays on two Illumina Sentrix-Mouse 6 Whole Genome Expression Beadchips (Illumina Inc., San Diego, CA). Illumina expression arrays were processed on a fee-for-service basis at the Cincinnati Children’s Hospital Medical Center Microarray Core Facility in Cincinnati, OH. Expression was quantified with an Ilumina Bead Scanner. Quality control and gene expression analysis were performed using Illumina BeadStudio v1.5.0.34. Illumina Sentrix-Mouse 6 Whole Genome Expression Beadchip. Signal intensities corresponding to each probeset were evaluated for similarity in mean expression values. Mean and variance of normalized expression values were similar for the two chips (12 individual microarrays) with the exception of a single microarray used to analyze one of the D2 samples. This sample was excluded from further analysis. The signal intensities were then normalized using the Rank Invariant normalization method, which is recommended for evaluating gene expression across similar chips. Differences in gene expression were identified using the Illumina Custom differential expression algorithm, which accounts for: 1) sequence specific biological variation; 2) nonspecific biological variation; and 3) technical error. The data were filtered by removing probesets that were absent across all chips and considered differentially expressed if the Illumina Custom differential expression algorithm generated a mean p-value of less that 0.05 (Illumina diffscore ≤13 or >13).

### Quantitative real-time PCR

Identical numbers 200,000 of cells from each of the four strains, as well as *Slit2*-GFP and GFP control cells, were sorted via flow cytometry. Total RNA from these cells was extracted and reverse transcribed into cDNA using Taqman Reverse Transcription Reagents (Applied Biosystems, Foster City, CA) and stored at −20 °C. Real-time PCR reactions were performed in TaqMan Universal PCR MasterMix with pre-made primer and probe mixes for mouse genes *Slit2, QDPR, GPR125, Robo4*, and endogenous *GAPDH* control (Applied Biosystems, CA) according to manufacturer’s instructions. PCR reactions were set up according to manufacturer’s instructions using TaqMan^®^ universal PCR master mix (PN 4304437). Analyses of gene expression were performed in single reporter assays in an ABI PRISM 7700 sequence detection system (PE Biosystems, Foster City, CA).

### Western blots

Cell samples were lysed at a concentration of 2 × 10^7^ cells/ml in protein lysis buffer containing: 10 mM Tris pH7.5, 50 mM NaCl, 30 mM sodium pyrophosphate, 50 mM NaF, 5 μM ZnCl2 and 1% Triton X-100, 2.8 ug/ml aprotinin (Sigma), 1 mM phenylmethylsulfonyl fluoride (Sigma), 1 mM sodium vanadate (Na3VO4) 1 ug/ml pepstatin, and 1 μg/ml leupeptin (Oncogene Research, MA). Lysate was incubated on ice for 30 min, and then centrifuged at 15,000 × g for 10 min to remove debris. The resulting supernatant was then aliquoted and stored at −80 °C. For Western blot, protein lysates were thawed and mixed with running buffer and a reducing agent (Novex, San Diego, CA, per manufacturer’s instructions) and heated at 95 °C for 5 min. Samples were then analyzed by denaturing PAGE (Novex, 10% bis-Tris gel) using the equivalent of 4 × 10^5^ cells per lane. Following electrophoresis, samples were electro-transferred onto immunobilon-P membranes (Millipore, Bedford, MA), which were subsequently blocked and probed with polyclonal rabbit anti-*Slit2* Ig-G antibody at a 1:3000 dilution. Primary antibodies were detected using alkaline phosphatase-conjugated secondary antibodies (Santa Cruz Biotechnology) and electro-chemifluorescent reagent (Pharmacia Biotech) according to the manufacturer’s instructions. Blots were visualized using a Molecular Dynamics STORM 860 system and Imagequant software. Following the detection and quantification of anti-*Slit2* antibody, immunobilon-P membrane was sequentially stripped in 40% methanol and the buffer containing 100 mm ß-mercaptoethanol, 2% sodium dodecyl sulfate and 62.4 mM Tris-HCl to remove electro-chemifluorescent reaction product and antibodies, respectively. The stripped membrane was re-probed with anti-actin antibody (Sigma) at 1:500,000 dilution and detected as described previously^20^.

### Retroviral transduction of primary mouse bone marrow cells

The retroviral vector, Sfbeta 91, served as a control and the backbone for cloning of *Slit2* cDNA. It contained the 5′-long terminal repeat derived from myeloproliferative sarcoma virus (MPSV) and a 3′-long terminal repeat derived from spleen focus forming virus (SFFV). The internal ribosomal entry site sequence derived from the encephalomyocarditis virus was used for simultaneous translation of gene insert and the gene for enhanced GFP. The *Slit2* cDNA sequence was cloned upstream of the IRES of the Sfbeta91 vector (MPSV-IRES-GFP-SFFV) to create a recombinant *Slit2*-carrying vector (MPSV-Lxn-IRES-GFP-SFFV). The *Slit2* cDNA was amplified from hematopoietic cells using *Slit2* forward primer: 5′ATTTGCGGCCGCAAGATGAGTGGCATTGGC3′ and *Slit2* reverse primer: 5′GGCCTCGAGTTAGGAGGCACATCTCGCGCA3′. Production of high-titer helper-free retrovirus was carried out by standard procedure in ectotropic Phoenix packaging cells as described previously^20^.

### Infection of primary murine bone marrow cells

Primary mouse bone marrow cells were transduced as previously described^20^. Briefly, bone marrow cells were harvested from mice treated 4 days previously with 150 mg/kg body weight 5-fluorouracil (5-FU; Sigma, St. Louis, MO) and cultured for 24 h in Dulbecco’s modified eagle’s medium supplemented with 10% FBS, 1% penicillin/streptomycin (Gibco Technologies, Carlsbad, CA), 50 ng/mL recombinant mouse stem cell factor, 10 ng/mL mouse interleukin 6 (mIL-6), and 10 ng/mL mouse interleukin 3 (mIL-3; R&D Systems, Minneapolis, MN). The cells were then harvested and slowly spread on the top surface of the membrane of a Transwell insert (Corning Incorporated Life Sciences, Acton, MA) at a density of 2 × 10^6^ cells per well. The viral supernatants were added to the Transwell inserts along with 4 μg/ml of polybrene and were cultured with cells for a further 48 h. The viral supernatant was changed 3 times during this period of time. Retrovirally-transduced, i.e. GFP positive, bone marrow cells were flow cytometrically sorted using a FACSAriaII (Becton-Dickinson) and used for CAFC assay and transplantation assays.

### Functional analysis of transduced cells

GFP marker was used to select transduced hematopoietic cells. GFP positive cells infected with the empty Sfbeta vector and the *Slit2*-containing vector were plated in limiting dilutions on confluent layers of FBMD-1 stromal cells for CAFC analysis. CAFC analysis was performed as previously^20^ described to measure the effects of *Slit2* expression on HSC and HPC numbers. Competitive repopulation assays with GFP positive cells were performed by co-transplantation of 2 × 10^5^ GFP^+^ cells with equal numbers of CD45.1 helper cells to two groups of lethally irradiated mouse recipients (10 recipients of GFP cells and 10 recipients of *Slit2*-GFP cells). Relative engraftment was determined by measuring the level of GFP positive cells in the peripheral blood of each recipient mouse 4–16 weeks post-transplant. In the limiting-dilution competitive repopulation assay as described previously^20^, graded numbers (6,000; 20,000; 60,000) of B6 GFP+ or B6 *Slit2* GFP+ cells (CD45.2) were admixed with a radio-protective dose (2 × 10^5^) of competitor cells (CD45.1) and injected intravenously into lethally irradiated (900 Gy) CD45.1 recipient mice. Relative engraftment was determined by measuring the level of GFP positive cells in the peripheral blood of each recipient mouse 12 weeks post-transplant as described above. The frequencies of long-term HSCs (CRU) were calculated from the proportions of negative recipients in each cell dose group, in which <1% of the circulating B, T and myeloid cells were regenerated by CD45.2 “test” stem cells, by using L-Calc software (StemCell Technologies Inc., Vancouver, BC).

### Mapping of eQTL for Slit2 in BXD mice

We queried gene expression data in hematopoietic stem cells of 23 BXD recombinant inbred strains using the UMCG Hematopoietic Stem Cells Illumina database in Genenetwork (http://genenetwork.org/webqtl/main.py) to map regulatory elements governing the expression of *Slit2* in these strains. Data for eight Illumina probes was available through in this database. We selected Illumina probe ILM1940037 which corresponded to the probe identified as differentially expressed in our microarray analysis and performed Interval mapping across the entire genome to identify regulators of *Slit2* expression or eQTL.

### Statistical analysis

Data were analyzed by either Student t-test with P < 0.05 (two-tail), or one-way ANOVA. All experimental methods were run in accordance with relevant guidelines.

## Additional Information

**Accession code:** GEO Accession# GSE21896.

**How to cite this article**: Waterstrat, A. *et al*. Quantitative trait gene *Slit2* positively regulates murine hematopoietic stem cell numbers. *Sci. Rep.* **6**, 31412; doi: 10.1038/srep31412 (2016).

## Supplementary Material

Supplementary Information
