# Supplementary material for: Quantitative trait gene Slit2 positively regulates murine hematopoietic stem cell numbers
Source: Sci Rep. 2016 Aug 9;6:31412. doi: 10.1038/srep31412 (PMC4977545; doi:10.1038/srep31412)
Supplement: Supplementary Information [file srep31412-s1.pdf]

Quantitative trait gene *Slit2* positively regulates murine hematopoietic stem cell numbers

Amanda Waterstrat, Kyle Rector, Hartmut Geiger, and Ying Liang

Supplementary Table 1. Hematopoetic progenitor cells (HPCs) and peripheral blood cell counts in Chr5 congenic and background mouse strains.

Mouse strains	HPC numbers		Peripheral blood cell accounts		
	CAFC day7 per femur (± 1 SEM)	CAFC day21 per femur (± 1 SEM)	Leukocytes ($x10^3$/ul ± 1 SD)	Erythrocytes ($x10^6$/ul ± 1 SD)	Platelets ($x10^5$/ul ± 1 SD)
B6	20016 ±1721	4608 ± 702	5.43 ± 1.56	9.78 ± 0.92	11.37 ± 1.21
B.D Chr5	28554± 1395	4782 ± 990	5.06 ± 1.70	9.91 ± 0.44	11.74 ± 1.21
D2	24512 ± 2130	12370 ± 1129	7.37 ± 1.08	10.04 ± 0.55	10.98 ± 0.83
D.B Chr5	25266± 3300	7220 ± 598*	6.68 ± 1.53	9.79± 0.29	10.70 ± 0.96

HPC numbers are represented by absolute numbers of CAFC day7 and day21 per femur. Cell counts for peripheral blood leukocytes, erythrocytes and platelets were performed in congenic and background mice (n ≥ 8 for each strain). Data shown are the average values ± 1 SEM (CAFC) and ±1 SD (peripheral blood) and compared among 4 strains by using one-way ANOVA analysis. No significant differences were revealed between Chr5 congenic and their respective background strains except that CAFC day21 number in D.BChr5 congenic mice was significantly lower than D2 background mice (* p=0.02).

Supp. Fig. 1

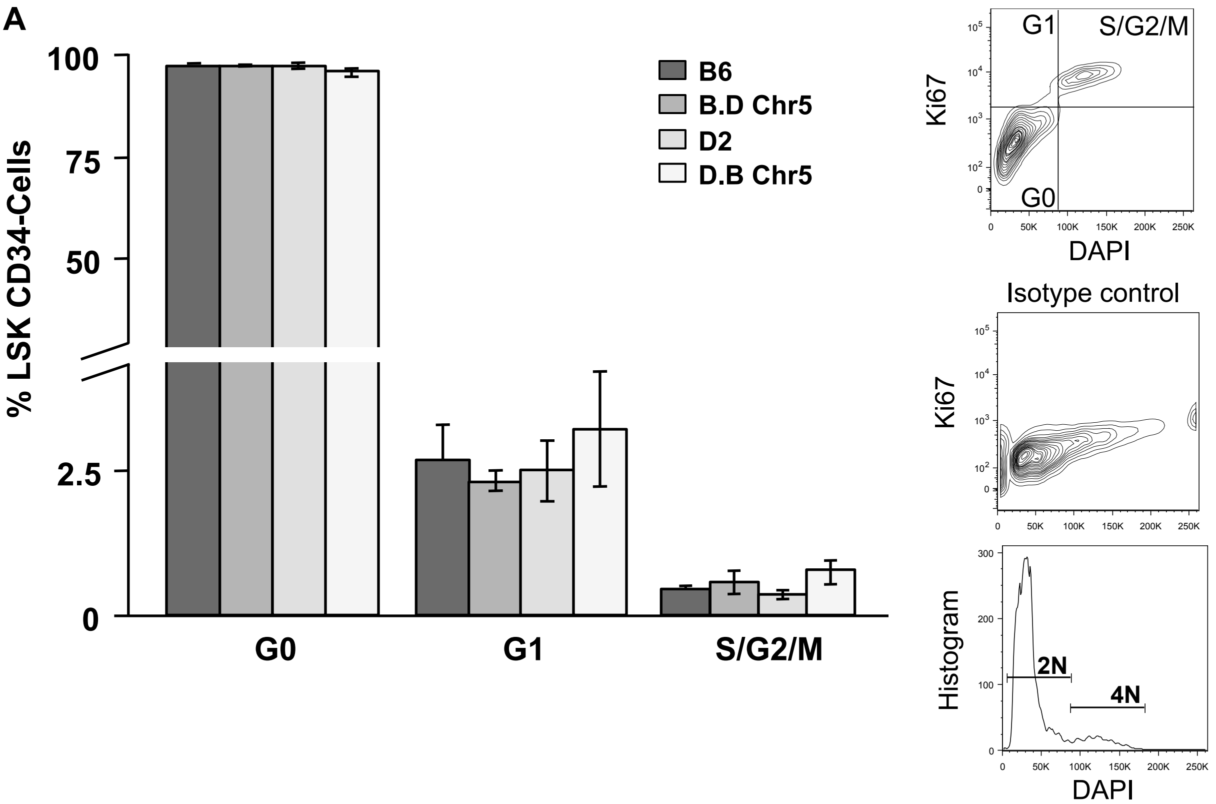
A
% LSK CD34-Cells
100
75
50
2.5
0
G0
G1
S/G2/M
B6
B.D Chr5
D2
D.B Chr5
G1
S/G2/M
G0
Ki67
DAPI
Isotype control
Ki67
Histogram
2N
4N
DAPI

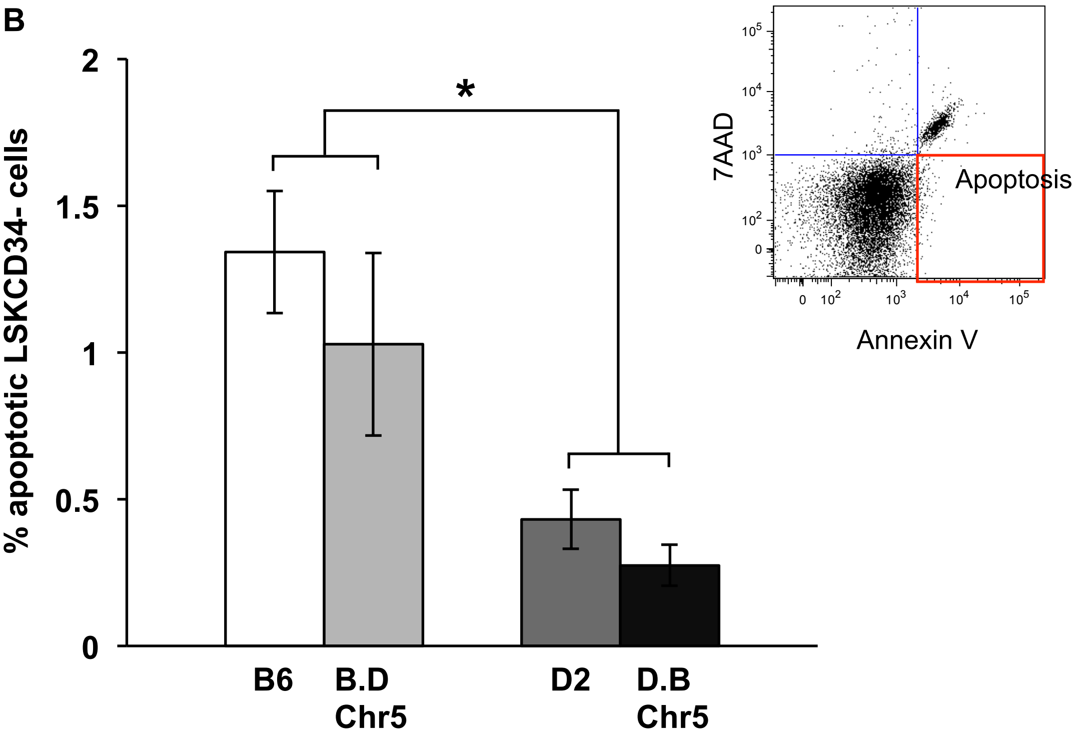
B
% apoptotic LSKCD34- cells
2
1.5
1
0.5
0
*
B6
B.D Chr5
D2
D.B Chr5
7AAD
Apoptosis
Annexin V

Supplementary Figure 1. Chr5 QTL does not affect cell cycle and apoptosis of HSCs.

(A) Chr5 QTL does not affect cell cycle of HSCs. Flow cytometric analysis of cell cycle showed no difference in the frequencies of each phase of cell cycle between Chr congenic and background HSCs. Representative FACS plots showing the G0 (Ki67- and DAPI-), G1 (Ki67+ and DAPI-), and S/G2/M (Ki67+and DAPI+) cell cycle phases in immunophenotypcially-defined HSCs (Lin-Sca1+c-Kit+CD34- cells) (top panel). Ki67 isotype control (middle panel) and histogram of DAPI staining (bottom panel) showing DNA content (2N and 4N) are representatively shown. The frequencies were presented as the average ± SD of 6 measurements from 2 independent experiments. (B) Chr5 QTL does not affect apoptosis of HSCs. Flow cytometric analysis of apoptosis showed no difference in the percentage of apoptosis between Chr congenic and their respective background HSCs although HSCs with B6 background are significantly higher than those with D2 background. Representative FACS plots showing Annexin V+ 7AAD- apoptotic cells (labeled in red rectangle). Presented data are the average ± SD of 2 independent experiments, each performed with 3 mice (n=6). $*p<0.05$
